# Voxel Normalization in LDCT Imaging: Its Significance in Texture Feature Selection for Pulmonary Nodule Malignancy Classification: Insights from Two Centers

**DOI:** 10.3390/diagnostics16020186

**Published:** 2026-01-07

**Authors:** Chen-Hao Peng, Jhu-Fong Wu, Chu-Jen Kuo, Da-Chuan Cheng

**Affiliations:** 1 Department of Biomedical Imaging and Radiological Science, China Medical University, Taichung 404328, Taiwan; u112202701@cmu.edu.tw; 2 Bioinformatics and Biostatistics Core, Centre of Genomic and Precision Medicine, National Taiwan University, Taipei 106319, Taiwan; 3 Department of Radiology, Ditmanson Medical Foundation Chia-Yi Christian Hospital, Chiayi 60002, Taiwan; 12669@cych.org.tw

**Keywords:** lung nodule, machine learning, deep learning, auto contouring fusion, computer-aided diagnosis

## Abstract

**Background:** Lung cancer is the leading cause of cancer-related mortality globally. Early detection via low-dose computed tomography (LDCT) can reduce mortality, but its implementation is challenged by the absence of objective diagnostic criteria and the necessity for extensive manual interpretation. Public datasets like the Lung Image Database Consortium often lack pathology-confirmed diagnoses, which can lead to inaccuracies in ground truth labels. Variability in voxel sizes across these datasets also complicates feature extraction, undermining model reliability. Many existing methods for integrating nodule boundary annotations use deep learning models such as generative adversarial networks, which often lack interpretability. **Methods:** This study assesses the effect of voxel normalization on pulmonary nodule classification and introduces a Fast Fourier Transform-based contour fusion method as a more interpretable alternative. Utilizing pathology-confirmed LDCT data from 415 patients across two medical centers, both machine learning and deep learning models were developed using voxel-normalized images and attention mechanisms, including transformers. **Results:** The results demonstrated that voxel normalization significantly improved the overlap of features between datasets from two different centers by 64%, resulting in enhanced selection stability. In the ROI-based radiomics analysis, the top-performing machine-learning model achieved an accuracy of 92.6%, whereas the patch-based deep-learning models reached 98.5%. Notably, the FFT-based method provided a clinically interpretable integration of expert annotations, effectively addressing a major limitation of generative adversarial networks. **Conclusions:** Voxel normalization enhances reliability in pulmonary nodule classification while the FFT-based method offers a viable path toward interpretability in deep learning applications. Future research should explore its implications further in multi-center contexts.

## 1. Introduction

In recent years, advancements in medical technology and oncology have contributed to a decline in annual mortality rates associated with cancer. Nevertheless, lung cancer (LC) remains a significant public health challenge, consistently reporting the highest incidence and mortality rates as indicated in the 2024 World Cancer Statistics [1]. In Taiwan, LC has been the leading cause of cancer-related fatalities for over the past decade [2]. Projections for 2024 anticipate that mortality from LC will be approximately threefold more significant than that from colorectal cancer, which ranks third, underscoring the considerable lethality of this malignancy [1].

One widely utilized lung cancer screening modality is low-dose computed tomography (LDCT) [3]. Lung nodules are typically detected in LDCT images when more significant than 3 mm in diameter. Nodules of this size and, more important, are generally visible and can be evaluated for further assessment, although smaller nodules may also be detected under optimal imaging conditions. For precise clinical contexts, guidelines may vary, and follow-up imaging may be recommended for nodules below this size.

In lung cancer studies, the “nodule spectrum” refers to the variety of lung nodules identified in imaging studies. This spectrum includes differences in size, shape, margins, and density. Nodules can range from very small to large and may exhibit smooth or irregular edges, from soft to complex in density. The growth patterns of nodules over time are also important for evaluation. Understanding this spectrum helps clinicians assess the risk of lung cancer and determine appropriate management strategies. For instance, one of the spectra can be classified into solid, part-solid, and ground-glass nodules (GGN) [4].

Several critical factors are assessed in the evaluation of lung nodules for potential malignancy. Nodule size is paramount; those exceeding 3 cm in diameter exhibit a higher probability of being malignant. Margin characteristics are also significant, as smooth, well-defined edges generally indicate benign lesions, while spiculated or irregular margins heighten suspicion of malignancy. The growth rate of the nodule is a crucial consideration; rapid enlargement is typically associated with malignancy, whereas stable nodules are often benign. Additionally, the density of the nodule plays a role, with solid nodules generally carrying a greater risk of cancer compared to ground-glass opacities. The pattern of calcification can aid in risk stratification, and patient-specific factors—including age, smoking history, and the presence of symptoms such as cough or weight loss—further inform the diagnostic process [4]. However, it is non-trivial to summarize these factors as a likelihood in cancer malignancy classification.

Despite the recognized utility of these biomarkers, a notable limitation exists regarding their effectiveness in enabling radiologists to reach unequivocal diagnostic conclusions [5]. Additionally, the diagnostic process is considerably impacted by the subjective interpretation of radiologists, leading to variability in diagnostic outcomes among practitioners with varying experience levels. Such diagnostic ambiguity often leads radiologists to recommend a biopsy to obtain definitive results. However, this approach carries the drawback of a significant false positive rate, increasing unnecessary invasive procedures [6,7]. However, with ongoing advancements in research, there is optimism for developing more reliable and precise diagnostic tools that may enhance diagnostic accuracy and consistency.

Significant research has been conducted on developing computer-aided diagnosis (CAD) systems, aiming to improve the accuracy of diagnosis in two main areas of lung cancer screening: nodule detection [8] and malignancy classification. Since this study focuses on nodule malignancy classification, we do not discuss nodule detection here.

The LIDC-IDRI dataset [9] is a popular dataset that primarily includes detailed annotations related to lung nodules, including their presence, size, and characteristics as assessed by radiologists. While it provides some information regarding the assessment of nodules, it does not explicitly label them as malignant or benign. Instead, the dataset includes radiologist assessments of the likelihood of malignancy, which is often based on subjective interpretations and consensus ratings but may not definitively classify each nodule’s malignancy status. Researchers typically use this information to develop and evaluate predictive models for nodule malignancy, such as [10,11,12,13,14].

Relying exclusively on the LIDC-IDRI dataset for diagnosing nodule malignancy presents certain limitations. This dataset lacks biopsy-confirmed pathological verification; instead, malignancy labels are derived from subjective assessments by radiologists, which may introduce bias in the training and evaluation of CAD models. To overcome this challenge, our study gathers LDCT data from patients with suspected lung cancer at local institutions, ensuring that all subjects have undergone biopsies for definitive pathological confirmation.

Another common challenge in relevant research utilizing open datasets is the reliance on unprocessed CT images for developing CAD systems. In real-world clinical settings [15], CT scans encounter variability in equipment settings, resulting in discrepancies in pixel spacing and slice thickness among patients. This variability results in non-uniform voxel sizes, which can negatively affect the comparability of extracted radiomic features and the generalization ability of machine learning (ML) models. To enhance the applicability and effectiveness of these systems, regardless of whether deep learning (DL) or ML techniques are employed, it is crucial to use normalized images with uniform parameters. Previous studies have addressed this issue, reaching a consensus on the importance of geometrically normalizing images before their utilization [16,17,18,19].

Moreover, the studies utilizing the LIDC-IDRI database for nodule classification face another challenge in managing contours delineated by four radiologists. Some studies only use contours from one radiologist [20], potentially missing insights from others. In contrast, others employ Generative Adversarial Networks (GANs) [21] to integrate all four, which can make models less interpretable and reduce clinical trust. This affects the consistency of classification outcomes. In this study, we will tackle this issue and demonstrate our solution to the problem.

In summary, this study has four primary objectives:

1. To collect two-center data with pathological verification for a reliable CAD system to differentiate between benign and malignant nodules.
2. To examine how voxel normalization affects nodule classification.
3. To create an intuitive contour fusion method for clinicians to merge contours from different radiologists.
4. To investigate distinct diagnostic paradigms—including feature-driven machine learning (radiomics) and data-driven deep learning methods—as the basis for the CAD system.

## 2. Materials and Methods

LDCT image data and their corresponding pathological confirmations are collected from two local centers: (a) Kaohsiung Veterans General Hospital (KVGH) and (b) Chia-Yi Christian Hospital (CYCH).

(a) The dataset includes 160 malignant and 81 benign pulmonary nodules (PNs) from 241 patients approved by the hospital’s Institutional Review Board (IRB number VGHKS18-CT5-09, date: 3 January 2018). Each nodule’s malignancy status was confirmed through pathological biopsy. Imaging was conducted using modalities from TOSHIBA, SIEMENS, GE MEDICAL SYSTEMS, and Philips. LifeX software (version 6.2.0, 2018, [22]) facilitated DICOM image reading and annotation in various planes. The recorded ROIs were saved as near-raw raster data (NRRD) files. Due to technical issues, 16 cases with annotation errors (3 benign and 13 malignant) were excluded. Feature extraction was performed on reconstructed images rather than raw scans [18].
(b) The dataset comprises 174 patients, including 78 benign cases (101 benign nodules) and 96 malignant cases (120 malignant nodules), approved by the hospital’s Institutional Review Board (IRB number IRB2022096, date: 16 November 2022). Each nodule’s malignancy status was confirmed through pathological biopsy. Imaging was conducted using various modalities: 104 patients with Siemens, 8 with GE Medical Systems, 11 with Toshiba, 2 with Canon Medical Systems, and 49 with Philips. All participants underwent imaging in Helical mode at 120 KVP. 122 patients received iodine-based contrast media, while 52 were scanned without contrast agents. LIFEx software (Version 6.2.0, 2018) was used to annotate regions of interest (ROIs), delineated independently by a skilled radiologist and a radiographer. The recorded ROIs were saved as NRRD files, with original CT files converted to NRRD format using SimpleITK.

The data formats are outlined: CT images are in int16 format, computations are in float64, and masks are binary. In radiomics, each bin contains 25 CT numbers. In deep learning, each CT image is transformed into a positive integer format within the range of [0, 255] using a lung window (window width: 1500, window level: −600).

### 2.1. General Frameworks for Dataset (A)

**Figure 1:**
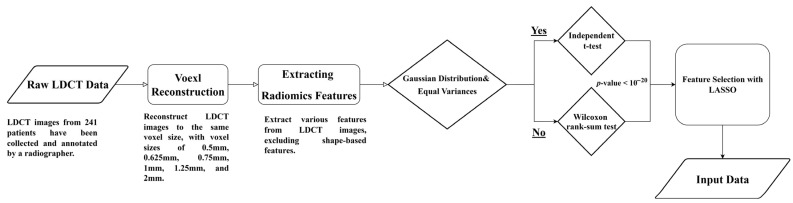
Flowchart (a) for LDCT Image Analysis and Radiomics Feature Extraction (Dataset A).

Figure 1 illustrates the flowchart for analyzing LDCT images and extracting radiomics features. The detailed steps are described as follows.

1. Raw LDCT Data: Collect LDCT images from KVGH.
2. Voxel Reconstruction for Geometric Normalization: Standardize images to defined isotropic voxel sizes (side-length 1.5 mm).
3. Radiomics Features Extraction: Obtain various features from the LDCT images while excluding shape-based features.
4. Statistical Testing:
  (1) Conduct tests for Gaussian distribution and equal variances.
  (2) If the criteria are met, perform an independent *t*-test; otherwise, use the Wilcoxon rank-sum test.
  (3) Determine statistical significance with a *p*-value threshold of less than (10^20^).
5. Feature Selection with LASSO: Apply LASSO (Least Absolute Shrinkage and Selection Operator) for feature selection on the dataset (a).

This methodology allows for efficient processing of LDCT data, with a focus on rigorous statistical validation and feature selection.

### 2.2. General Frameworks for Dataset (B)

**Figure 2:**
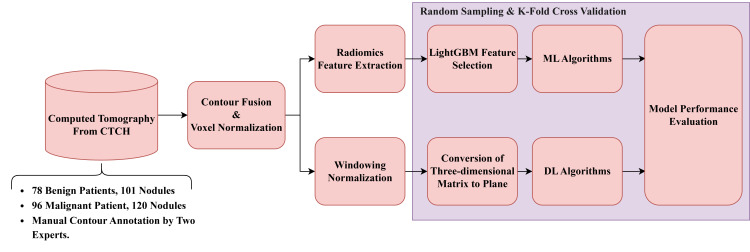
Flowchart (b) for LDCT Image Analysis and Radiomics Feature Extraction (Dataset B).

Figure 2 illustrates the general framework for dataset (B). The detailed procedures are described as follows.

1. Raw LDCT Data: Collect LDCT images from CYCH.
2. Voxel Reconstruction for Geometric Normalization: Standardize images to defined isotropic voxel sizes (side-length 1.5 mm).
3. Radiomics Features Extraction: Obtain various features from the LDCT images while excluding shape-based features. Before feature extraction, some filters and transformations are applied to generate comprehensive images, details of which are described in Section 2.5.
4. Feature Selection and Classification
  (1) With LightGBM: Apply Light Gradient Boosting Machine (LightGBM) for feature selection on radiomics features of dataset (b), followed by different ML classifiers.
  (2) With neural networks: Apply different NNs.

Note: (1) and (2) are independent; they generate different results.

### 2.3. Isotropic Voxel Normalization on CT Images

To improve the consistency and effectiveness of machine learning models for identifying the malignancy of PNs, the study standardizes pixel size (PS) and slice thickness (ST) across all LDCT images. This is performed using isotropic voxel normalization with bicubic interpolation [23], ensuring uniform spatial resolution and achieving three-dimensional isotropy. This research also examines the effects of different voxel sizes (side lengths from 0.5 to 2 mm) on model performance and the characteristics of extracted features. To rigorously evaluate the stability of the models, we performed statistical significance tests using the Wilcoxon rank-sum test. Specifically, we compared the performance metrics of each isotropic voxel size against a baseline derived from original, non-normalized images. This analysis ensures that the performance variations observed at different spatial resolutions are statistically significant compared to the unadjusted baseline.

### 2.4. Nodule Contour Fusion

The Fast Fourier Transform (FFT) is crucial for contour fusion of nodules from different expert annotations. This process is conducted on 2D binary images (0 for absence and 1 for presence of a nodule). The 2D FFT converts these binary images into their spectral forms, which are then averaged to create a merged spectrum. The inverse Fast Fourier Transform (IFFT) reverts the average spectrum to the spatial domain. Finally, the absolute values of the reversed images are processed. Instead of selecting an arbitrary threshold, we conducted a sensitivity analysis to determine the optimal threshold value. We evaluated threshold parameters ranging from 0.4 to 0.8 to identify the value that best balances the inclusion of nodule features with the exclusion of non-tumorous background tissues. Based on the performance metrics (detailed in Section 3), a threshold of 0.7 was identified as the optimal operating point to finalize the fusion. The contour fusion process is detailed in Equations (1) and (2) for the case of two experts.

(1) $$ROI_{fusion}=ABS\left(IFFT\left(\frac{FFT(ROI_{first\;expert})+FFT(ROI_{second\;expert})}{2}\right)\right)$$

(2) $$ROI_{fusion}\left(i,j\right)=\{\begin{array}{c} 1,\;\;ROI_{fusion}\left(i,j\right)\geq 0.7\\ 0,\;\;ROI_{fusion}\left(i,j\right)<0.7 \end{array}$$

The fused contour is then used for feature extraction. Notably, this process can also be conducted in 1D form, where the coordinates of nodule contours are the inputs and outputs.

### 2.5. Feature Extraction and Selection

Radiomics [24] is a well-known technique focused on extracting diverse features from medical images, encompassing first-order, shape-based, and texture features. Our study employs the pyradiomics library [25] to extract these features from contour-defined ROIs. Before the extraction process, we apply various filters to the raw ROIs, including Wavelet filters [26], the Laplacian of Gaussian filter [27], and several transformations such as Square, Square Root, Logarithm, Exponential, and Gradient, along with the Local Binary Pattern technique [28]. These preprocessing steps aim to enhance the dataset by generating additional images for more comprehensive feature extraction.

The extensive process results in 2120 features. To address the challenges of high dimensionality, we utilize the LightGBM [29] for feature selection, which differs from our previous method [18]. LightGBM is an efficient gradient-boosting framework known for its speed and effectiveness in handling large datasets, making it ideal for balancing limited computational resources and training time. Another significant advantage of LightGBM is its ability to rank features by their importance during training, based on each feature’s contribution to decision tree construction and its effect on model accuracy. Specifically, we first employ Spearman’s test to evaluate linear relationships among all features, removing those with collinearity exceeding 0.9. The remaining features are then used to train the LightGBM model, allowing us to identify and select the 38 most effective features for further model development.

### 2.6. Classifiers in Machine Learning (ML)

Six ML algorithms are investigated, including Logistic Regression, Multilayer Perceptron (MLP), Random Forest, Linear Discriminant Analysis (LDA), LightGBM, and CatBoost [30].

### 2.7. CT Image Preparation for Neural Networks

To prepare input images for convolutional neural networks (CNNs), we employ a windowing technique on all CT images using a lung window [31], characterized by a window width of 1500 and a window level of −600. This adjustment normalizes the CT image values to the range of [0, 255], enhancing lung nodular visibility. The transformation function is illustrated in Equation (3). Subsequently, we extract patches with dimensions of 64 × 64 × 9 from each CT image sequence, ensuring that the nodules are centered within these patches. These nine patches are then flattened into a single image with dimensions of 192 × 192 × 1, serving as the input to the 2D neural networks

(3) $$\mathrm{Windowing\_img}\left(i,j\right)=\frac{\mathrm{original\_img}\left(i,j\right)-\left(WL-\frac{WW}{2}\right)}{WW}$$

where *WW* = Window Width, *WL* = Window Level.

We utilize 2D input rather than 3D input in our deep learning models. This decision is based on the fact that 3D input requires significantly higher computational resources than its 2D counterparts [32]. The 3D format entails longer training time and necessitates a larger memory capacity for the model. Consequently, this research does not employ 3D images as the input. However, we test 3D deep learning models to compare performance between 2D and 3D inputs. For the 3D model, we use a matrix of dimensions 9 × 64 × 64 × 1 as the input, and the model is ‘3D ResNet101’, which is described in Section 2.9.

### 2.8. 2D Neural Networks and DL Algorithms

In our research, we employ various DL algorithms, exploring a range of CNN architectures, including VGG16 [33], ResNet101 [34], InceptionNet [35], and ConvNeXt [36]. Additionally, we investigate two multi-modal models: EVA02 [37] and Meta Transformer [38]. Furthermore, we comprehensively evaluate six contemporary self-attention models, which include the Dual Attention Vision Transformers (DaViTs) [39], Vision Outlooker for Visual Recognition (VOLO) [40], Swin Transformer V2 [41], Phase-Aware Vision MLP (Wave-MLP) [42], LeViT [43], Dilated Neighborhood Attention Transformer (DINAT) [44], and Masked Image Modeling with Vector-Quantized Visual Tokenizers (BEIT v2) [45]. These models have been selected for their innovative contributions to processing complex visual data through advanced attention mechanisms and architectural designs. Our objective in testing such a diverse set of self-attention models is to assess their efficacy in classifying lung nodule malignancy.

Each DL model is initially pre-trained on the ImageNet1000 dataset [46] and subsequently fine-tuned through transfer learning [47] using our in-house dataset. All training processes adhere to the same early stopping criterion [48], which halts training if the validation loss does not improve for more than ten epochs, indicating model convergence. All models utilize the Adadelta optimizer [49], maintain a cyclic learning rate [50] (with a maximum learning rate of 0.1 and a minimum learning rate of 0.00001), and employ Binary Focal Loss with an alpha value of 2 [51]. Our experimentation with all models proceeds using the configuration of an RTX 3090 alongside an i9-7900X, with a setup that includes PyTorch 2.1.0 and Ultralytics 8.2.8.

### 2.9. 3D ResNet101

The 3D ResNet101 [52,53] represents an evolution of the traditional ResNet architecture, tailored explicitly for processing 3D data. In contrast to the standard 2D ResNet, the 3D ResNet101 is optimized for handling volumetric data such as video frames or medical imaging scans [52]. This model maintains the deep residual network structure, connecting layers with identity mappings to address the vanishing gradient problem that often occurs in intense networks. The “101” denotes the network’s depth, which comprises 101 layers. In the 3D variant, the 2D convolutional layers are replaced with 3D convolutions, allowing the model to capture three-dimensional information. This feature makes it particularly effective for tasks such as action recognition in videos, 3D object recognition, and volumetric medical image analysis. We selected this model for comparison instead of others because it is also used for pulmonary micronodule malignancy risk classification [52].

### 2.10. Model Performance Validation

We implemented ten-fold cross-validation to rigorously assess the performance of the various models. Crucially, to prevent data leakage and ensure the generalizability of our results, the dataset partitioning was performed strictly at the patient level rather than the nodule level. This ensures that all nodules belonging to the same patient are assigned exclusively to either the training or the testing set in each iteration, thereby preventing the model from recognizing patient-specific features across splits. The patient cohort was divided into ten subsets; nine were used for training (employing transfer learning), and one was reserved for testing. This procedure was repeated ten times, with each subset serving as the test set once. The final performance is reported as the average across all ten folds. We evaluated the models using Balanced Accuracy, Weighted Sensitivity, Weighted Precision, Weighted F-score, and Weighted AUC, as defined in Equations (4)–(7), ensuring a comprehensive and robust assessment of effectiveness.

(4) $$Balanced\;Accuracy=\frac{1}{k}\sum_{i=0}^{k}\frac{TP_{i}}{\left(TP_{i}+FN_{i}\right)};$$

(5) $$Weighted\;Precision=\frac{\sum_{i=0}^{k}w_{i}\times TP_{i}}{\sum_{i=0}^{k}w_{i}\times \left(TP_{i}+FP_{i}\right)};$$

(6) $$Weighted\;F1\;score=\frac{\sum_{i=0}^{k}w_{i}\times \left(\frac{2\times TP_{i}}{2\times TP_{i}+FP_{i}+FN_{i}}\right)}{\sum_{i=0}^{k}w_{i}};$$

(7) $$Weighted\;AUC=\frac{\sum_{i=0}^{k}w_{i}\times AUC_{i}}{\sum_{i=0}^{k}w_{i}},$$

where *TP*, *FP*, and *FN* denote true positive, false positive, and false negative, respectively.

### 2.11. Understanding Object Recognition: Model Interpretability and Visibility

Gradient-weighted Class Activation Mapping (Grad-CAM) [54] is a prominent technique designed to enhance the interpretability of deep learning models by generating heatmaps that illustrate the regions of an input image that most influence the model’s class predictions. This method involves computing the gradients of the class score concerning the input image, which is then backpropagated to the model’s final convolutional layer. By weighting and combining these gradients, Grad-CAM produces a feature map that indicates the model’s focus areas within the input. This visual representation can be overlaid on the original image to highlight regions that capture the model’s attention, thereby facilitating an understanding of whether it relies on relevant image features for classification. This increases transparency and not only assists in validating the model’s decision-making process but exposes potential biases or shortcomings. In this study, Grad-CAM is effectively utilized to elucidate the model’s attention mechanisms, confirming its accurate identification of nodule characteristics in medical imaging.

## 3. Results

### 3.1. Nodule Contour Fusion

**Figure 3:**
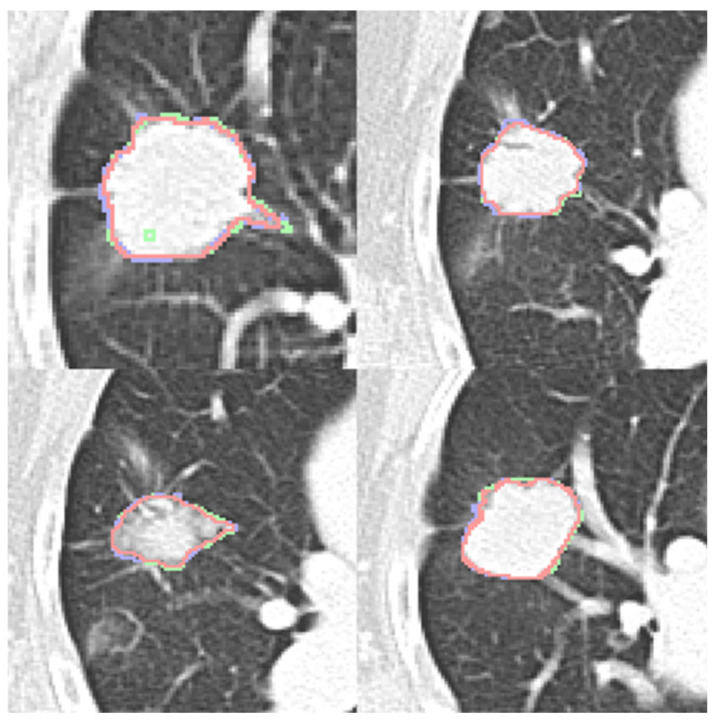
Four contour fusion results. Three contours are superimposed on a raw patch. The blue and green lines illustrate contours meticulously delineated by two human experts. The red line represents the contour fusion, which aligns the nodule areas from the two experts, providing a refined depiction.

Four expert contours exist in the LIDC-IDRI dataset and two in the dataset (b). Four qualitative contour fusion results are demonstrated in Figure 3. Three contours are superimposed on the raw patch. The blue and green colors represent two manual contours, while the red color indicates the fusion result. Minor inconsistencies are observable. Notably, there is a manual error in Figure 3 (top left), represented in green, where an extra point is erroneously included. However, the fusion result does not reflect this mistake. The proposed contour fusion technique effectively mitigates sharp variations exhibited by both experts, such as protrusions or indentations, thereby addressing the gaps left by incomplete annotations.

### 3.2. Feature Extraction and Selection

**Figure 4:**
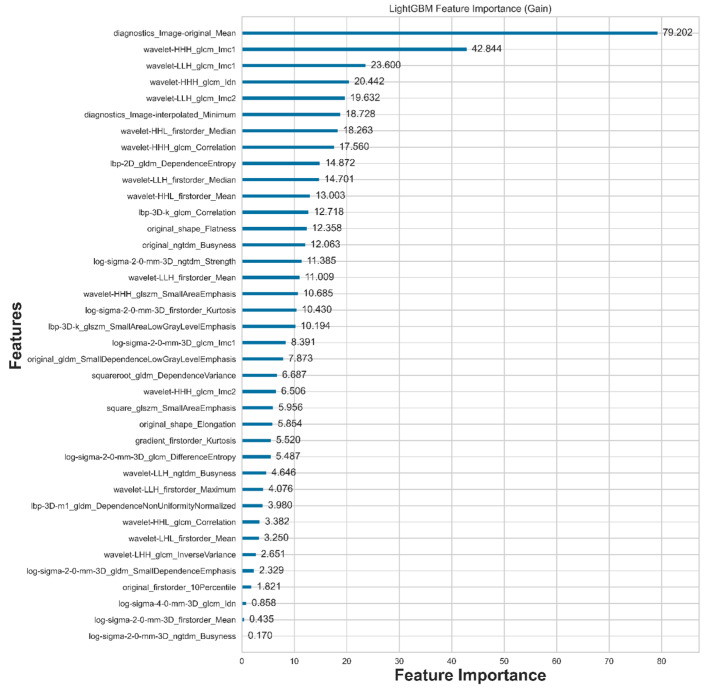
Features selected by LightGBM and their corresponding importance to nodule malignancy classification.

A total of 2120 features are extracted using Radiomics. After feature selection with LightGBM, only the 38 most compelling features are retained, as illustrated in Figure 4. From the table, we can observe that there are 12 statistical features, 2 shape features, and 24 texture features, with eight statistical features ranked at the top.

Compared to the results of our previous study published in [18], there are 11 features selected by LASSO. We have created a table that reveals the feature intersection between dataset (a) and dataset (b) based on the same voxel normalization but with different feature selection methods, as shown in Table 1. We found that 7 out of 11 features appear again, resulting in a ratio of 63.6%.

### 3.3. Classifiers in Machine Learning

**Figure 5:**
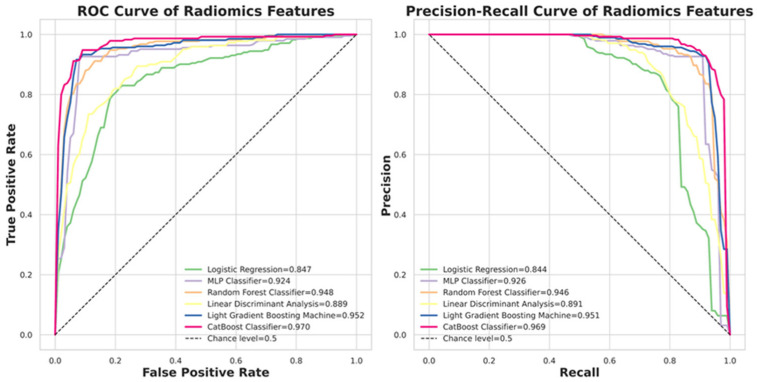
The performance of six classifiers. CatBoost is the top classifier in the nodule malignancy classification using the dataset (b) in this study.

Figure 5 presents the ROC and Precision–Recall curves for the six evaluated classifiers. CatBoost achieved the highest ROCAUC of 0.968, while Logistic Regression recorded the lowest at 0.847. Detailed quantitative performance metrics for all models are summarized in Table 2.

**Figure 6:**
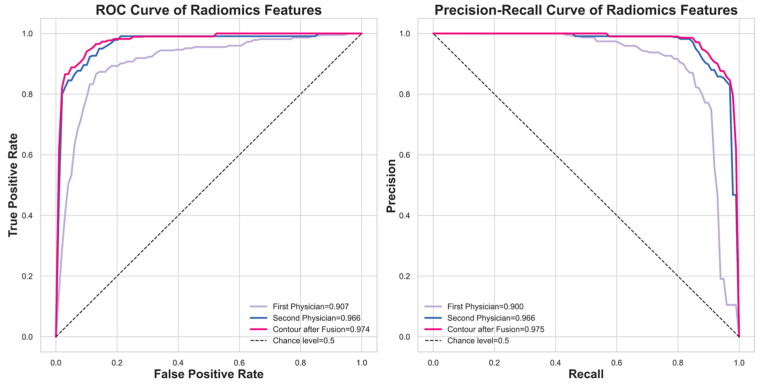
The model performance comparison between using the fused contour and using the experts’ contour.

Figure 6 compares the classification performance using the fused contour versus individual expert contours via ROC and Precision–Recall curves. The fused contour achieved the highest performance with a ROCAUC of 0.974. In comparison, the contours delineated by the first and second experts yielded ROCAUC values of 0.907 and 0.966, respectively.

### 3.4. CT Image Preparation for Neural Networks

**Figure 7:**
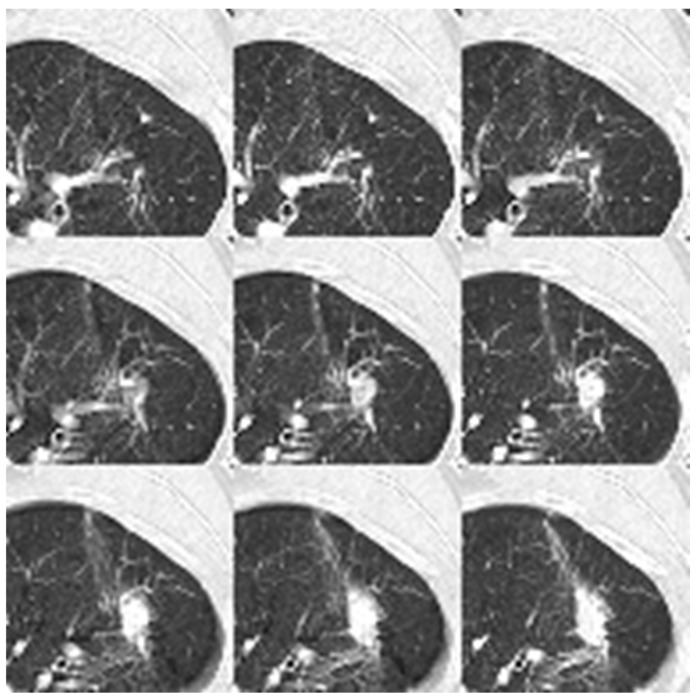
Nine patches are flattened to a dimension of 192 × 192 for the 2D neural network input. The data format is uint8.

Figure 7 visualizes the processed input for the 2D neural networks. The image displays the aggregation of nine individual 64 × 64 patches, which are flattened into a single 192 × 192 composite plane to serve as the model input.

Table 3 summarizes the quantitative performance of the evaluated deep learning models. The 3D ResNet101, DaViT, and BEIT v2 architectures emerged as the top performers, each achieving identical metrics with an accuracy of 0.985, sensitivity of 0.972, and precision of 1.000. In terms of discrimination capability, the attention-based models (DaViT and BEIT v2) reached an AUC of 0.999, marginally surpassing the 0.998 AUC of 3D ResNet101 regarding model complexity; 3D ResNet101 utilizes significantly fewer parameters (~11 million) compared to DaViT (~50 million) and BEIT v2 (~86 million). However, despite this parameter efficiency, the 3D architecture necessitates higher computational resources due to the processing requirements of volumetric inputs compared to the 2D models.

Table 4 presents a performance comparison of the proposed models relative to other models published in recent years, highlighting various metrics, including accuracy, sensitivity, precision, F1-score, and AUC. The results indicate that the deep learning models developed in this study—specifically 3D ResNet101, DaViT, and BEIT v2—each achieved an accuracy of 0.985, with sensitivity and precision scores of 0.972 and 1.000, respectively. Additionally, these models achieved F1-scores of 0.986 and AUC values ranging from 0.998 to 0.999, showcasing their strong performance in nodule malignancy classification regarding handcrafted feature analysis. The CatBoost model demonstrates solid effectiveness with an accuracy of 0.926, a sensitivity of 0.925, and a precision of 0.927, alongside an F1-score and AUC of 0.926 and 0.968, respectively. It is important to note that this performance was achieved using ROI-based texture features, which inherently contain less spatial information compared to the full-spectrum patch-based inputs utilized by the DL models.

Among other recent studies, Kang et al. [10] and Saihood et al. [12] exhibit high accuracy, with the former achieving an accuracy of 0.984 and the latter reaching 0.987. However, the AUC values for Saihood et al. are not reported. Conversely, Halder et al. [11] yield an AUC of 0.993, demonstrating competitive performance. Overall, this table illustrates the competitive positioning of the proposed models against both historical and contemporary benchmarks in nodule malignancy classification.

To investigate the impact of spatial resolution on deep learning performance, we conducted an ablation study using the BEIT v2 model across five isotropic voxel sizes ranging from 1.5 mm to 0.625 mm. The quantitative results are detailed in Table 5. The model achieved its highest performance at the 1.5 mm resolution with an accuracy of 0.985 ± 0.012 and a statistically significant *p*-value of 0.034. Similarly, the 1.25 mm and 1.0 mm resolutions maintained high accuracies above 0.979 with significant *p*-values of 0.032 and 0.027, respectively. At the 0.75 mm resolution, the accuracy decreased to 0.958 ± 0.028, while the *p*-value of 0.048 remained below the significance threshold. Finally, the 0.625 mm resolution yielded the lowest accuracy of 0.935 ± 0.042 with a *p*-value of 0.062, which exceeded the threshold for statistical significance.

### 3.5. Model Interpretability and Visibility

**Figure 8:**
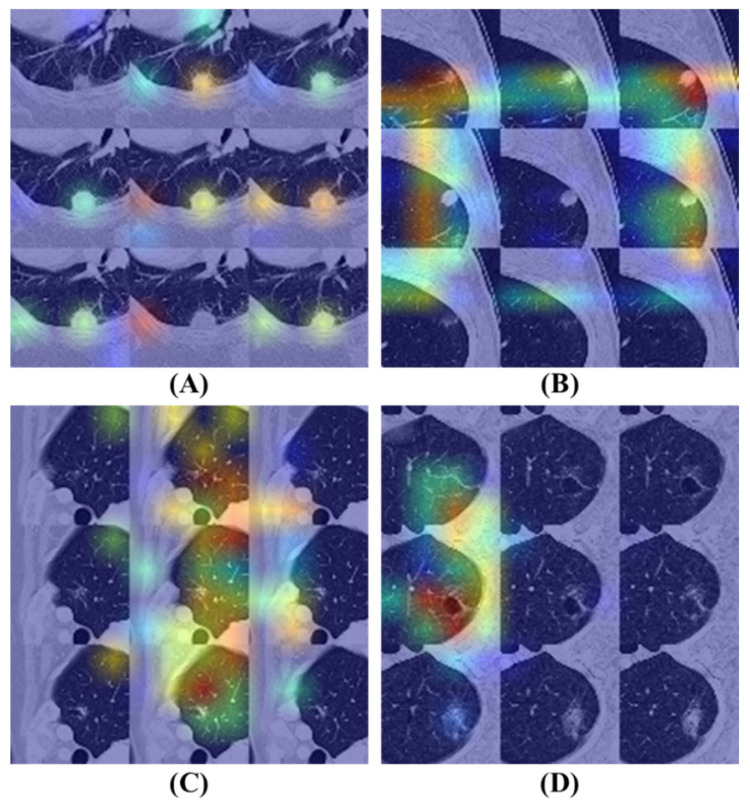
Grad-CAM visualizations of the BEIT v2 model’s attention mechanisms on flattened nodule patches. Warmer colors (red/yellow) indicate regions with higher influence on the classification decision. (**A**,**B**) Correctly classified instances where the model accurately focuses on the nodule texture while ignoring irrelevant background areas. (**C**,**D**) Misclassified cases illustrating potential confounders: (**C**) The model erroneously focuses on peripheral vascular structures rather than the nodule. (**D**) The model is distracted by surrounding emphysematous tissue, leading to an incorrect diagnosis.

Figure 8 illustrates the Grad-CAM results of the BEIT v2 model, providing insights into its attention mechanisms. Figure 8A,B display attention maps corresponding to cases where the model’s predictions are correct. In these examples, the model effectively identifies significant features within the nodule regions, demonstrating a focused approach by allocating minimal attention to non-relevant areas of the images.

In contrast, Figure 8C,D present attention maps for instances where the model’s diagnoses are incorrect. Despite the model concentrating on specific regions, the highlighted areas are inaccurate. Figure 8C reveals that the model disproportionately focuses on vessels located near the lung’s peripheral region, contributing to its erroneous diagnosis. Similarly, Figure 8D shows that the model allocates excessive attention to areas affected by emphysema within the lung, leading to an incorrect classification.

## 4. Discussion

**Figure 9:**
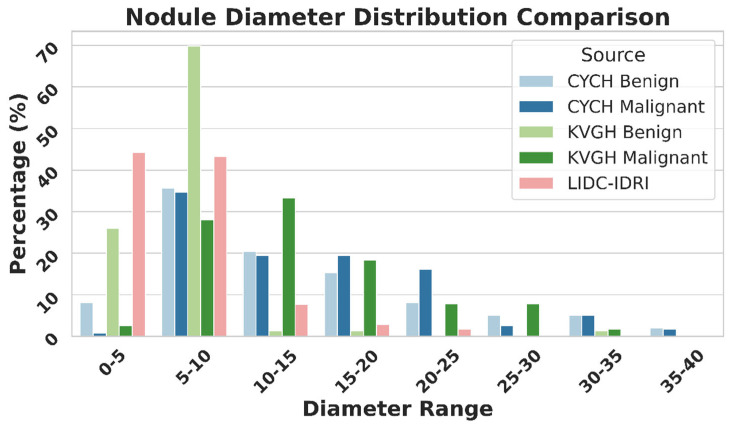
Nodule Diameter Distribution as Percentage by Source.

Figure 9 illustrates the differences in nodule size distribution between KVGH and CYCH, revealing that KVGH nodules are generally larger, while those from CYCH are smaller. Additionally, the benign and malignant nodules in CYCH exhibit a greater degree of overlap in size, further complicating classification. Nevertheless, 64% of the selected features overlap between the two datasets, underscoring the significance of voxel normalization in achieving consistent feature selection across varying distributions. This discrepancy also helps explain why the ML models in this study performed slightly less effectively than our previous research [18]. Since CYCH serves as the primary dataset and contains smaller nodules and more significant overlap between benign and malignant cases, the classification task is inherently more challenging, resulting in more incredible difficulty distinguishing between benign and malignant cases.

Furthermore, Figure 9 emphasizes the limitations of using the LIDC-IDRI dataset for training nodule classification models. Although the nodules in LIDC-IDRI exhibit considerable diameter overlap with those in CYCH and KVGH, they lack pathology-proven diagnoses and rely solely on the subjective malignancy ratings provided by radiologists. This reliance increases the risk of erroneous ground truth labels, which could lead to unreliable model performance when applied to datasets with confirmed diagnoses.

It is important to acknowledge that the comparison between ML and DL models reflects a fundamental difference in input modalities: ROI-based radiomics versus patch-based raw images. For the ML analysis, we deliberately focused on texture-based stability, explicitly excluding 14 geometric features (e.g., volume, size) and employing a coarser 25-bin configuration for CT numbers. While this approach ensured a rigorous evaluation of texture features across centers, it inherently limited the information available to the ML classifiers (e.g., CatBoost). In contrast, the patch-based DL models utilized inputs with full 256 grayscale levels and preserved spatial contexts. Consequently, the superior performance of the DL models should be attributed to their ability to leverage high-resolution, pixel-level information, whereas the ML models were restricted to discretized, aggregated texture descriptors within the ROIs.

While numerous studies have advocated for 3D ResNet architectures to capture volumetric spatial information, we demonstrate that 2D Transformer-based models (specifically DaViT and BEIT v2) achieve equivalent diagnostic performance (AUC ~0.999) without the prohibitive computational overhead associated with 3D networks. Traditional 2D CNNs often struggle with nodule classification due to their limited receptive fields; however, the self-attention mechanisms in Transformers effectively capture long-range dependencies across the entire image, mimicking the contextual understanding usually attributed to 3D models.

From a practical clinical perspective, selecting 2D Transformers over 3D architectures offers significant advantages for routine LDCT workflows. Although 3D ResNet101 contains fewer parameters, it necessitates processing volumetric data (9 × 64 × 64), which demands substantially higher video memory and floating-point operations (FLOPs) during inference. In contrast, 2D models require significantly lower computational resources, allowing for faster inference times and reducing the dependency on high-end, expensive GPU hardware. This efficiency is critical for integrating CAD systems into standard clinical workstations or PACS (Picture Archiving and Communication Systems), where hardware resources are often constrained. Therefore, the 2D Transformer-based approach provides a more scalable and cost-effective solution for large-scale lung cancer screening programs.

The challenges associated with ML-based approaches are evident. Manual contour delineation of nodules is time-consuming, and the feature selection can vary between different runs, leading to inconsistencies among researchers. Our findings emphasize that voxel normalization is essential in processing LDCT data, as 64% of the features selected in a previous study reemerged in this research, highlighting its significance in maintaining feature stability.

To further elucidate the impact of voxel normalization on deep learning architectures, our ablation study using BEIT v2 provides critical insights into the trade-off between spatial resolution and model stability. The observed performance plateau between 1.0 mm and 1.5 mm suggests that this resolution range optimally preserves the semantic features requisite for malignancy classification without introducing redundant information. Conversely, the degradation in accuracy coupled with a drastic increase in standard deviation at finer resolutions (specifically 0.625 mm) indicates that excessive spatial detail likely introduces high-frequency noise and reconstruction artifacts. Crucially, the loss of statistical significance at 0.625 mm (*p* = 0.062), despite a substantial drop in mean accuracy, statistically corroborates the model’s severe instability and susceptibility to overfitting. This finding parallels our observations in the ML analysis, reinforcing that 1.5 mm isotropic voxel normalization is a pivotal preprocessing step for ensuring robustness in both radiomics and deep learning pipelines.

One limitation of this research is the relatively small dataset, comprising only 415 patients from two medical centers. While utilizing pathology-confirmed LDCT data represents a significant strength, a more extensive dataset obtained from multiple centers would validate the findings more robustly. Additionally, merging annotations from various experts for nodule boundaries poses a considerable challenge. Some studies have explored using GANs for contour fusion; however, these models can be complex for clinicians to interpret. As an alternative, we propose an approach based on the FFT that mathematically integrates boundaries while preserving shape details. This method achieves performance comparable to that of GANs while being more interpretable. Furthermore, the computational speed of FFT is significantly greater than that of GANs, and it does not require expensive hardware such as GPUs, making it a more cost-effective solution that is better suited for clinical applications.

To determine the optimal error tolerance for this contour fusion technique, we performed a sensitivity analysis on the FFT thresholding parameter, ranging from 0.4 to 0.8. As presented in Table 6, the threshold of 0.7 was identified as the optimal operating point, achieving the highest accuracy of 0.926 and an F1-Score of 0.926. Our analysis reveals a clear trade-off: thresholds below 0.7 (0.4–0.6) exhibit a slight degradation in performance (accuracy ~0.912–0.918). While these lower thresholds yield higher sensitivity by generating broader contours, they inadvertently incorporate non-tumorous background tissues, thereby reducing precision. Conversely, increasing the threshold to 0.8 results in a sharp decline in performance, with accuracy dropping to 0.865 and sensitivity to 0.784. This degradation suggests that an overly strict threshold excludes peripheral nodule textures essential for malignancy characterization. Therefore, the 0.7 threshold represents the critical balance point that maximizes feature stability and classification power.

Our findings underscore the pivotal role of voxel normalization, emphasize the limitations of the LIDC-IDRI dataset, and illustrate the effectiveness of FFT-based annotation fusion. Future research should investigate voxel normalization’s impact on deep learning models and compare various contour integration techniques to refine pulmonary nodule classification further.

## 5. Conclusions

This study delved into the impact of voxel normalization and an automatic contour fusion CAD system. Our experimental findings revealed that normalizing the voxel size to 1.5 mm resulted in optimal CAD performance. We observed a high level of consistency in the texture features extracted and selected from data obtained from different centers. Using only 11 features and SVM, we achieved an impressive accuracy of 0.9596 and an AUC of 0.9855 among 241 patients. Furthermore, by employing the Fast Fourier Transform to fuse contours drawn by different experts and leveraging the BEIT V2 model, we attained an accuracy of 0.9848 and an AUC of 0.9994 on a dataset of 221 nodules. It is worth noting that all nodules utilized in this study were pathologically verified as the gold standard, underpinning the development of a robust and reliable CAD system for clinical practitioners.

## Figures and Tables

**Table 1:** 11 features LASSO [18] selected appear in the current study and their corresponding ranks.

Feature Name	Type	Rank
original_gldm_SmallDependenceLowGrayLevelEmphasis	Texture	Rank 21
log-sigma-2-0-mm-3D_glcm_DifferenceEntropy	Texture	Rank 27
log-sigma-2-0-mm-3D_gldm_SmallDependenceEmphasis	Texture	Rank 34
log-sigma-3-0-mm-3D_glszm_ZonePercentage	Texture	X
lbp-2D_gldm_DependenceNonUniformityNormalized	Texture	X
lbp-3D-m1_gldm_DependenceNonUniformityNormalized	Texture	Rank 30
lbp-3D-m2_gldm_DependenceNonUniformityNormalized	Texture	X
log-sigma-2-0-mm-3D_firstorder_Mean	First-order	Rank 37
lbp-3D-m1_firstorder_Skewness	First-order	X
wavelet-LLH_firstorder_Mean	First-order	Rank 16
wavelet-LHL_firstorder_Mean	First-order	Rank 32

Where X means there is no match.

**Table 2:** Models performance using different evaluation metrics.

	Accuracy	Sensitivity	Precision	F1-Score	ROCAUC
LR	0.802	0.795	0.810	0.797	0.847
RF	0.887	0.887	0.886	0.887	0.949
LDA	0.817	0.811	0.823	0.813	0.890
MLP	0.907	0.906	0.908	0.907	0.924
LightGBM	0.919	0.919	0.918	0.919	0.953
Catboost	0.926	0.925	0.927	0.926	0.968

LR: Logistic Regression, RF: Random Forest, LDA: Linear Discriminant Analysis, MLP: Multilayer Perceptron. Ten-fold cross-validation.

**Table 3:** Comparison of NN Models’ performance using different evaluation metrics.

	Accuracy	Sensitivity	Precision	F1 Score	ROCAUC
EVA02	0.531	0.778	0.550	0.645	0.607
VGG16	0.924	0.973	0.897	0.934	0.950
InceptionNet	0.924	0.918	0.944	0.939	0.967
Meta Transformer	0.956	0.918	1.000	0.957	0.977
LeViT	0.924	0.861	1.000	0.926	0.978
ResNet101	0.940	0.973	0.921	0.946	0.981
Swin Transformer V2	0.955	0.944	0.972	0.959	0.989
DINAT	0.985	0.973	1.000	0.986	0.992
Wave-MLP	0.985	0.972	1.000	0.986	0.995
ConvNeXt	0.955	0.917	1.000	0.957	0.997
VOLO	0.939	1.000	0.900	0.947	0.998
3D ResNet101 *	0.985	0.972	1.000	0.986	0.998
DaViT	0.985	0.972	1.000	0.986	0.999
BEIT v2	0.985	0.972	1.000	0.986	0.999

Ten-fold cross-validation. * Only 3D ResNet101 has 3D patches input, the rest models have 2D patches input.

**Table 4:** Performance comparison of the proposed models with other models published in recent years.

	Accuracy	Sensitivity	Precision	F1-Score	AUC
Kang et al. 2017 [10]	-	0.984	-	-	0.990
Dey et al. 2018 [13]	0.904	-	-	-	0.954
Mehta et al. 2021 [14]	-	-	-	-	0.939
Saihood et al. 2022 [12]	0.987	0.984	-	-	-
Halder et al. 2022 [11]	0.961	0.968	-	-	0.993
Hsiao et al. 2023 [18]	0.959	0.961	0.963	0.961	0.985
CatBoost (ours)	0.926	0.925	0.927	0.926	0.968
3D ResNet101 (ours)	0.985	0.972	1.000	0.986	0.998
DaViT (ours)	0.985	0.972	1.000	0.986	0.999
BEIT v2 (ours)	0.985	0.972	1.000	0.986	0.999

**Table 5:** Performance comparison of the BEIT v2 model across different isotropic voxel sizes.

Voxel Size	Accuracy	Sensitivity	Precision	F1-Score	AUC	*p*-Values
1.5	0.985 ± 0.012	0.972 ± 0.015	1.000 ± 0.006	0.986 ± 0.013	0.999 ± 0.001	0.034
1.25	0.983 ± 0.014	0.970 ± 0.018	0.998 ± 0.005	0.984 ± 0.015	0.998 ± 0.002	0.032
1	0.979 ± 0.018	0.965 ± 0.021	0.994 ± 0.009	0.979 ± 0.019	0.995 ± 0.004	0.027
0.75	0.958 ± 0.028	0.938 ± 0.035	0.980 ± 0.025	0.959 ± 0.030	0.982 ± 0.012	0.048
0.625	0.935 ± 0.042	0.910 ± 0.051	0.962 ± 0.045	0.935 ± 0.048	0.968 ± 0.025	0.062

**Table 6:** Performance sensitivity of the CatBoost model to different FFT fusion thresholds.

Threshold	Accuracy	Sensitivity	Precision	F1-Score	AUC
0.4	0.912	0.948	0.88	0.913	0.955
0.5	0.915	0.942	0.891	0.916	0.958
0.6	0.918	0.938	0.902	0.92	0.961
0.7	0.926	0.925	0.927	0.926	0.968
0.8	0.865	0.784	0.952	0.86	0.895

## Data Availability

The data presented in this study are available on request from the corresponding authors. The data are not publicly available due to privacy and ethical restrictions imposed by the Institutional Review Board of Ditmanson Medical Foundation Chia-Yi Christian Hospital.

## Abbreviations

The following abbreviations are used in this manuscript:

LDCT	Low-Dose Computed Tomography
PN	Pulmonary Nodule
NSCLC	Non-Small Cell Lung Cancer
SCLC	Small Cell Lung Cancer
AC	Adenocarcinoma
SCC	Squamous Cell Carcinoma
GGN	Ground-Glass Nodule
CAD	Computer-Aided Diagnosis
ML	Machine Learning
DL	Deep Learning
CNN	Convolutional Neural Network
FFT	Fast Fourier Transform
ROI	Region of Interest
LASSO	Least Absolute Shrinkage and Selection Operator
LIFEx	Local Image Feature Extraction (software)
LightGBM	Light Gradient Boosting Machine
MLP	Multilayer Perceptron
LDA	Linear Discriminant Analysis
AUC	Area Under the Curve
ROC	Receiver Operating Characteristic
Grad-CAM	Gradient-weighted Class Activation Mapping
BEiT	Bidirectional Encoder Representation from Image Transformers
DaViT	Dual Attention Vision Transformer
VOLO	Vision Outlooker
DINAT	Dilated Neighborhood Attention Transformer
VGG	Visual Geometry Group
ResNet	Residual Network
MetaFormer	Meta Transformer
EVA	Enhanced Visual Attention
NCHC	National Center for High-performance Computing
KVGH	Kaohsiung Veterans General Hospital
CYCH	Chia-Yi Christian Hospital
IRB	Institutional Review Board
